# YOLO-PEL: The Efficient and Lightweight Vehicle Detection Method Based on YOLO Algorithm

**DOI:** 10.3390/s25071959

**Published:** 2025-03-21

**Authors:** Zhi Wang, Kaiyu Zhang, Fei Wu, Hongxiang Lv

**Affiliations:** School of Electronic and Electrical Engineering, Shanghai University of Engineering Science, Shanghai 201602, China; m320122318@sues.edu.cn (Z.W.); m325121514@sues.edu.cn (K.Z.); m325122241@sues.edu.cn (H.L.)

**Keywords:** object detection, YOLO, lightweight, multi-scale detection

## Abstract

YOLOv8-PEL shows outstanding performance in detection accuracy, computational efficiency, and generalization capability, making it suitable for real-time and resource-constrained applications. This study aims to address the challenges of vehicle detection in scenarios with fixed camera angles, where precision is often compromised for the sake of cost control and real-time performance, by leveraging the enhanced YOLOv8-PEL model. We have refined the YOLOv8n model by introducing the innovative C2F-PPA module within the feature fusion segment, bolstering the adaptability and integration of features across varying scales. Furthermore, we have proposed ELA-FPN, which further refines the model’s multi-scale feature fusion and generalization capabilities. The model also incorporates the Wise-IoUv3 loss function to mitigate the deleterious gradients caused by extreme examples in vehicle detection samples, resulting in more precise detection outcomes. We employed the COCO-Vehicle dataset and the VisDrone2019 dataset for our training, with the former being a subset of the COCO dataset that exclusively contains images and labels of cars, buses, and trucks. Experimental results demonstrate that the YOLOv8-PEL model achieved a mAP@0.5 of 66.9% on the COCO-Vehicle dataset, showcasing excellent efficiency with only 2.23 M parameters, 7.0 GFLOPs, a mere 4.5 MB model size, and 176.8 FPS—an increase from the original YOLOv8n’s inference speed of 165.7 FPS. Despite a marginal 0.2% decrease in accuracy compared to the original YOLOv8n, the parameters, GFLOPs, and model size were reduced by 25%, 13%, and 25%, respectively. The YOLOv8-PEL model excels in detection precision, computational efficiency, and generalizability, making it well-suited for real-time and resource-constrained application scenarios.

## 1. Introduction

With the rapid advancement of science and technology and the accelerated pace of urbanization, the number of vehicles has significantly increased, leading to a series of traffic issues [1] that severely impact the road traffic environment. These problems not only threaten road safety but also affect traffic flow and the health of urban ecosystems.

Deep learning, with its unique advantages, has gradually become a key research direction in vehicle image target detection. Traditional vehicle recognition methods typically rely on human or machine vision [2], requiring traffic police or intelligent monitoring systems to identify vehicles through visual observation. However, on the data level, factors like significant lighting variations, noise interference, and disruption of light propagation in road environments, along with differences in vehicle appearance and movement states [3], often lead to numerous false positives and missed detections in detection models. On the algorithmic level, vehicle feature extraction in complex environments poses a primary challenge. Additionally, since vehicle detection algorithms are usually deployed on traffic monitoring devices, they require models with low parameter counts, minimal computation, and high real-time performance.

Advanced object detection technologies have demonstrated exceptional performance in improving detection efficiency, reducing costs, and minimizing the missed detection rate. These object detection techniques are primarily based on feature- and segmentation-based algorithms [4,5], which focus on characteristics such as target color, texture [6], and shape [7]. These techniques significantly enhance detection accuracy and real-time capability, meeting the practical needs of intelligent transportation systems in complex road environments. Vehicle detection remains a challenging issue in the field of object detection [8].

To address this challenge, we propose the YOLOv8-PEL vehicle detection algorithm, based on the YOLOv8n network model, to enhance the accuracy and detection efficiency of vehicle target detection in real-time application scenarios. Experimental results demonstrate that YOLOv8-PEL achieved a mAP@0.5 of 66.9% on the COCO-Vehicle dataset. Compared to the baseline YOLOv8n model, the parameters, GFLOPs, and model size of YOLOv8-PEL were reduced by 30%, 13%, and 25%, respectively, with an 11.1 increase in FPS. The primary contributions of this paper are as follows:

1. The PPA module is introduced into the backbone network to construct the C2F-PPA network module. The primary role of C2F-PPA is to effectively retain multi-scale features of the target using a parallel multi-branch strategy. Compared to the original C2F structure, C2F-PPA has an increased depth, which improves accuracy and enhances the model’s generalization capability.
2. The limitations of the original channel attention (CA) module were identified, such as its reliance on 1 × 1 convolutions, which restricts feature extraction capability, fails to fully capture complex spatial information, and performs inadequately in handling long-range dependencies. Therefore, the ELA mechanism is introduced to enhance feature selection and fusion capabilities through 1D convolutions and larger convolutional kernels (such as five or seven), improving feature extraction performance.
3. An Enhanced Local Attention Feature Pyramid Network (ELA-FPN) is proposed to address the multi-scale challenges in vehicle detection. ELA-FPN enhances feature selection and fusion capabilities through 1D convolutions and uses Group Normalization (GN) instead of Batch Normalization (BN), which improves the model’s generalization ability, reduces model size, and increases processing speed.
4. The Wise-IoUv3 loss function is used to optimize the model, reducing harmful gradients caused by extreme samples. By introducing gradient gain and a dynamic non-monotonic mechanism, the focus on regular anchor boxes is enhanced, which improves the accuracy and speed of bounding box regression.

## 2. Related Work

### 2.1. Traditional-Based Methods for Vehicle Detection

Traditional vehicle detection methods primarily rely on inherent features of vehicles, including color, symmetry, edges, texture, shadows, and taillights. The detection process typically unfolds in two stages: Hypothesis Generation (HG) and Hypothesis Verification (HV). During the HG phase, the system exploits the continuity and concentration of color to segregate vehicles from the background; it searches for regions of high symmetry in the image by detecting the symmetry at the rear end of vehicles; and it preliminarily determines the location of vehicles by extracting edge features such as contours and bumpers. Concurrently, it further extracts Regions of Interest (ROIs) by discerning the uneven texture features on the vehicle’s surface or by utilizing the shadow areas cast by vehicles in sunlight. In nocturnal environments, the red characteristic of taillights also serves as an effective cue. Following the HG phase, the HV phase validates the extracted ROIs to ascertain the presence of vehicle targets. Although traditional methods have certain advantages in feature extraction and detection speed, their effectiveness is susceptible to variations in environmental lighting and background interference. Hence, the fusion of multiple features, combined with more advanced modeling techniques such as machine learning or deep learning, can further enhance the accuracy and robustness of detection.

### 2.2. Machine-Learning-Based Methods for Vehicle Detection

With the advancement of computer technology, machine learning has emerged as a pivotal methodology in the field of vehicle detection. ML encodes images through manually designed features, transforming high-dimensional data into low-dimensional representations, and trains models to perform vehicle detection. Vehicle detection based on ML typically involves two critical steps: initially processing the input image to extract ROIs, followed by feeding the image features into a classifier for categorization and optimization. In feature extraction, the features must be easily recognizable and stable across variations in vehicle posture and type. Popular methods such as Histogram of Oriented Gradients (HOG) [9], which achieved success in pedestrian detection, have been widely adopted in vehicle detection and have inspired a variety of enhanced algorithms, such as HOG pyramid [10] and symmetric HOG [11]. Classifiers then differentiate vehicles from non-vehicles based on the extracted features. For instance, Support Vector Machines (SVM), Decision Trees (DT), and AdaBoost are commonly utilized classifiers. Ensemble learning, which improves accuracy by combining predictions from multiple classifiers, poses a challenge due to its high computational cost. Integrating feature extraction with classifier-based approaches (such as using shadows to extract ROIs and employing AdaBoost for detection) can effectively reduce computational resource consumption.

### 2.3. Deep-Learning-Based Methods for Vehicle Detection

In recent years, detection methods based on computer vision combined with deep learning have gradually become the mainstream algorithms in the field [12], eliminating the need for manual feature extraction [12]. Currently, object detection algorithms are primarily categorized into two types:

The first category consists of two-stage object detection algorithms, such as R-CNN [13], Fast R-CNN [14], Faster R-CNN [15], and Mask R-CNN [16]. These algorithms divide the object detection task into two phases: candidate box generation and candidate box classification. The advantage of two-stage detection algorithms lies in their ability to enhance detection accuracy, as the two-phase process allows for the precise localization of target objects. The candidate box generation in the first phase reduces the search space and provides candidate boxes with high recall rates, while the classification and regression in the second phase further refine the candidate boxes for precise localization and classification.

Although two-stage object detection algorithms can offer high detection accuracy in vehicle detection, they also have several notable drawbacks. Firstly, their inference speed is slow, mainly because it requires the generation of candidate regions followed by classification and regression for each region. This staged processing approach increases computational complexity and is not suitable for real-time detection tasks. Secondly, these algorithms demand significant computational resources, making them inefficient on resource-constrained embedded devices or mobile environments. Additionally, in high-density scenarios such as congested traffic or parking lots, two-stage algorithms are prone to false positives or missed detections in candidate regions, leading to reduced recall rates, and the complexity of the models poses challenges for optimization and deployment.

The second category includes one-stage object detection algorithms, such as SSD [17], YOLO [18,19,20,21,22,23], and RetinaNet [24]. One-stage detection algorithms perform object detection directly within the image, without the need for explicit candidate box generation and classification stages, hence offering superior detection speed compared to two-stage algorithms. Due to their excellent speed and high overall performance, one-stage object detection algorithms have become an important tool in the field of vehicle detection.

YOLOv3 is one of the significant versions of the YOLO model, followed by the widely applied YOLOv5, which was open-sourced by ultralytics. YOLOv5n achieves lightweight processing through model compression and pruning but falls short in multi-scale feature fusion; YOLOv7-tiny introduces a dynamic label assignment mechanism but has limited detection accuracy in complex scenarios. In 2023, ultralytics released the latest version in the YOLO series, YOLOv8, which incorporates numerous improvements in model architecture, training strategy, and performance optimization, resulting in a notable enhancement in both accuracy and speed.

Despite the superior detection accuracy and speed of the YOLOv8 model compared to the aforementioned one-stage detection methods, it has its limitations. Particularly in real-time application scenarios in the vehicle detection domain (such as autonomous driving and traffic monitoring), where the detection model is required to process rapidly while maintaining a certain level of accuracy. Hence, an improvement is needed to increase the detection speed of vehicle targets while achieving a balance with detection accuracy.

## 3. Methods

### 3.1. YOLOv8 Model

The network structure of YOLOv8 comprises four modules: Input, Backbone, Neck, and Head. The modular design ensures efficiency and stability in object detection, detailed as follows:

Input: The input images undergo Mosaic data augmentation to enrich the dataset. Moreover, an anchor-free strategy is employed to reduce the number of predicted boxes, thereby accelerating the Non-Maximum Suppression (NMS) process.

Backbone: This includes modules like Conv, C2f, and SPPF. The Conv module is responsible for operations like convolution, Batch Normalization, and SiLU activation on the input images. The C2f module allows for a richer flow of gradient information while remaining lightweight. The SPPF module extracts and encodes image features at different scales.

Neck: This comprises the FPN and PAN. FPN strengthens semantic features through a top-down propagation approach, while PAN enhances location features through a bottom-up propagation approach. The combination of FPN and PAN effectively fuses feature maps at different stages.

Head: A decoupled head strategy is adopted, separating the classification head from the detection head. The category and location information of the target are obtained based on feature maps of three different scales.

This model has five versions: n, s, m, l, x, all of which share a similar network model, with differences in network depth and width. Among these, the YOLOv8n network has the smallest depth and width. Considering the practical application scenarios of vehicle target detection, the YOLOv8n model was chosen for further improvement in this paper due to its simple network structure, minimal computational resource requirements, and fastest operation speed.

### 3.2. C2F-PPA Module

In YOLOv8, the C2f module is responsible for fusing high-level semantic features with low-level detail features to enhance detection accuracy. In object detection tasks, key information can be easily lost during multiple downsampling operations. To optimize vehicle detection tasks, this paper proposes the C2F-PPA module to address this issue. The C2F-PPA module incorporates an innovative network technique called the Parallelized Patch-Aware Attention (PPA) module [25]. This module enhances network performance and efficiency by replacing traditional convolution operations in the encoder and decoder.

**Figure 1:**
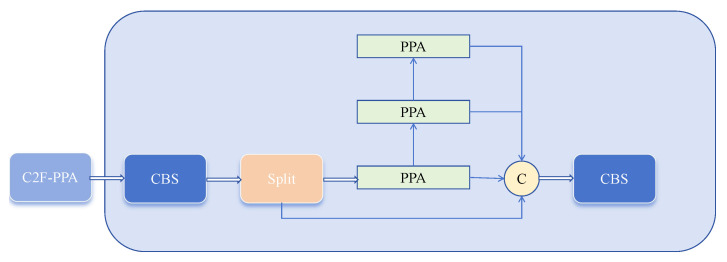
C2F-PPA module.

**Figure 2:**
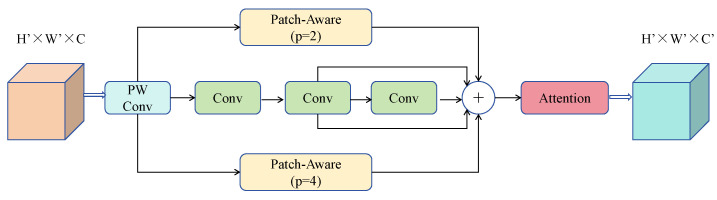
Parallelized Patch-Aware Attention module.

We improved the C2F structure within the Backbone by introducing the PPA module. As shown in Figure 1, we use the PPA module in the middle layers of the C2F structure to generate output instead of the traditional Bottleneck module. The main advantage of the PPA module lies in its multi-branch feature extraction strategy. As illustrated in Figure 2, PPA employs a parallel multi-branch approach, with each branch extracting features at different scales and levels, which helps capture multi-scale features of targets and thus enhances detection accuracy. In the modified C2F-PPA module, the input to the final layer includes multiple branches, forming a structure akin to a dense residual structure. This residual structure is advantageous for optimization and can improve accuracy by increasing network depth. The internal residual blocks use skip connections to mitigate gradient vanishing issues that arise in deep neural networks with increasing depth. Consequently, the C2F-PPA module, compared to the original C2F structure, has greater depth and improved accuracy.

Building on the original feature extraction mechanism, the improved C2F-PPA module adds receptive field attention, enhancing spatial feature extraction, and channel attention, which strengthens feature extraction from both spatial and channel dimensions. Additionally, the parallel multi-branch strategy of the C2F-PPA module effectively preserves the multi-scale features of the target objects.

To address this issue, we aimed to leverage the strengths of the C2F-PPA module to enhance the deep semantic feature extraction capability of CSPDarknet53. We replaced the original C2F module between the second and third output layers of CSPDarknet53 with our C2F-PPA module, aiming to enhance deep feature semantic extraction without compromising shallow feature details.

### 3.3. ELA-FPN

In YOLOv8’s feature fusion module, the PANet demonstrates significant advantages compared to the traditional FPN. PANet effectively enhances the fusion process across multi-scale features through its innovative bidirectional information flow mechanism. This mechanism not only facilitates the transfer of low-level detail information to higher layers but also ensures that high-level semantic information enhances the representation of lower-layer features. This advantage provides superior performance when processing images with rich detail and complex backgrounds. Although PANet has achieved considerable progress in multi-scale feature fusion, there is still room for improvement. Initially, PANet did not fully exploit its potential when handling large-scale feature maps, which may result in the loss of some critical information, ultimately affecting the overall performance of object detection. Additionally, some original information may be lost during the upsampling and downsampling processes.

To address these issues effectively, this study reconstructs YOLOv8’s feature fusion module based on the High-level Screening-feature Pyramid Network (HS-FPN) framework [26]. HS-FPN processes input feature maps with a CA module [27], utilizing the Sigmoid activation function to determine the weight of each channel and ultimately obtain the weight of each channel. The filtered feature maps are generated by multiplying these weight values with the corresponding scale feature maps, and then dimension matching is performed across different scales. Through a Selective Feature Fusion mechanism, high-level and low-level information from these feature mappings is integrated synergistically. This fusion yields features rich in semantic content, which aids in detecting subtle details within images, thereby enhancing the model’s detection capabilities.

In the original HS-FPN, we observed limitations in the CA module, such as the restrictive use of 1 × 1 convolutions that hinder its feature extraction capability and prevent it from fully capturing complex spatial information. Additionally, CA has limitations in handling long-range dependencies, especially within deeper network layers, and Batch Normalization sometimes adversely affects model generalization.

**Figure 3:**
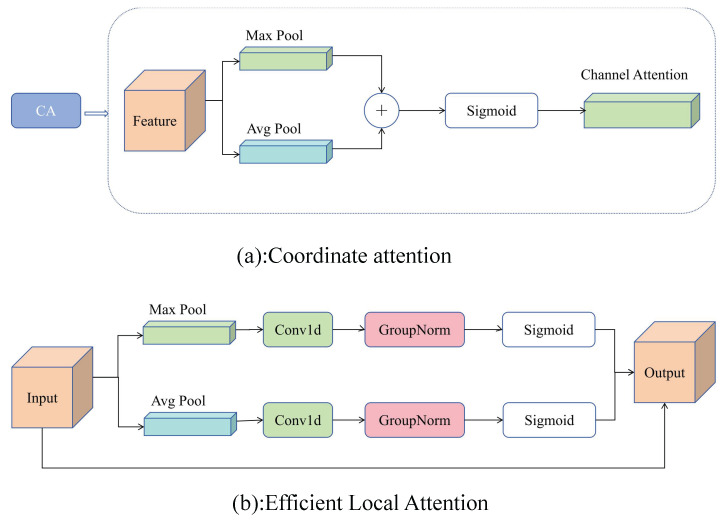
Comparison between ELA and CA.

**Figure 4:**
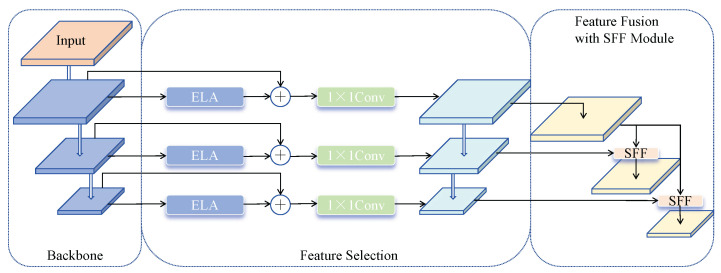
ELA-FPN.

To address these issues, we propose an ELA-FPN to optimize the feature fusion process. ELA-FPN replaces the CA module with an Enhanced Local Attention (ELA) mechanism [28] to handle multi-scale features more effectively. As shown in Figure 3, the ELA mechanism uses 1D convolutions to improve positional information along both horizontal and vertical axes. By incorporating larger convolution kernels (such as five or seven), ELA improves feature selection and fusion, allowing it to better handle long-range dependencies and enhance feature extraction. Additionally, ELA replaces Batch Normalization with Group Normalization, which enhances the model’s generalization capacity, particularly when processing diverse vehicle images. This adjustment allows the model to capture vehicle feature information more precisely, improving detection performance. The structure of ELA-FPN, as shown in Figure 4, consists of two main components: (1) a feature selection module and (2) a feature fusion module.

Feature Selection Module: The ELA module play important roles in this process. The ELA module initially processes the input feature map $f_{in}\in R^{C\times H\times W}$, where *C* represents the number of channels, *H* represents the height of the feature map, and *W* represents the width of the feature map.

To prompt the attention module to capture long-range interactions with precise positional information in space, we decompose global pooling into a pair of 1D feature encoding operations. Given the input *x*, we employ two pooling kernels with different spatial extents, (H,1) and (1,W), to encode each channel along the horizontal and vertical coordinate directions, respectively. Thus, the output at height h for the c-th channel can be expressed as:

(1) $$z_{c}^{h}\left(h\right)=\frac{1}{H}\sum_{0\leq i<H}x_{c}\left(h,i\right)$$

Similarly, the output at width w for the c-th channel can be represented as:

(2) $$z_{c}^{w}\left(w\right)=\frac{1}{W}\sum_{0\leq j<W}x_{c}\left(j,w\right)$$

The ELA enhances positional information in the horizontal and vertical directions through 1D convolution and processes these enhanced information using Group Normalization to generate positional attention maps $y_{h}$ and $y_{w}$:

(3) $$y_{h}=\sigma \left(Gn\left(F_{h}\left(z_{h}\right)\right)\right)$$

(4) $$y_{w}=\sigma \left(Gn\left(F_{w}\left(z_{w}\right)\right)\right)$$

where σ is the nonlinear activation function, $F_{h}$ and $F_{w}$ represent 1D convolutions with kernel sizes of five or seven. The final output of the ELA module *Y* is obtained through Equation (5):

(5) $$Y=x_{c}\times y_{h}\times y_{w}$$

By multiplying the positional attention map with the feature map at the corresponding scale, a filtered feature map is generated. Then, a 1 × 1 convolution is applied to reduce the number of channels for each scale feature map to 256, ensuring dimensional consistency across different scales.

Feature Fusion Module: The multi-scale feature maps generated by the Backbone network present high-level features with rich semantic information but relatively coarse target localization. In contrast, low-level features provide precise target location but contain limited semantic information. A common solution is to directly add the high-level features with the low-level features pixel-by-pixel after upsampling to enrich the semantic information of each layer. However, this technique only performs pixel-wise addition between feature layers without feature selection. To address this limitation, the Selective Feature Fusion (SFF) module is used.

**Figure 5:**
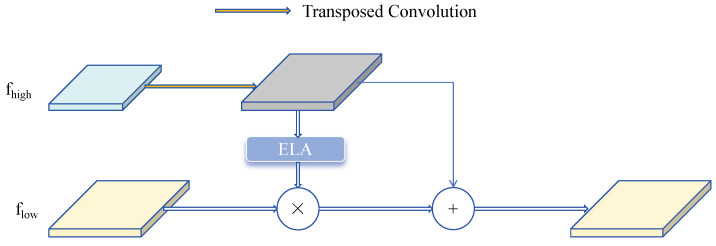
Selective Feature Fusion module.

The SFF module filters key semantic information embedded in low-level features using high-level features as weights and strategically fuses features. As shown in Figure 5, given the input high-level feature $f_{high}\in R^{C\times H\times W}$ and the input low-level feature $f_{low}\in R^{C\times H1\times W1}$, the high-level features are first expanded through transposed convolution with a stride of 2 and a kernel size of 3 × 3, obtaining feature size ${\hat{f}}_{high}\in R^{C\times 2H\times 2W}$. The ELA module converts high-level features into corresponding attention weights to filter low-level features, obtaining features of consistent dimensions. Finally, the filtered low-level features are fused with high-level features, enhancing the model’s feature representation, obtaining $f_{out}\in R^{C\times H1\times W1}$.

The enhanced feature representation significantly improves the model’s ability to detect fine features in vehicle images, thereby enhancing detection capability. ELA-FPN effectively addresses the multi-scale challenges in vehicle detection by introducing the Enhanced Local Attention mechanism and strategic feature fusion based on HS-FPN. This approach not only overcomes the limitations of the CA module but also significantly improves the accuracy and robustness of vehicle recognition tasks. By more comprehensively capturing vehicle feature information, ELA-FPN demonstrates great potential in practical applications.

### 3.4. Optimizing the Loss Function

The magnitude of the loss value reflects the discrepancy between the predicted value and the true value; the smaller the loss, the better the network’s regression capability. The IoU loss function is as shown in Equation (6):

(6) $$L_{\mathrm{IoU}}=1-IoU=1-\frac{W_{i}H_{i}}{S_{u}}$$

(7) $$S_{u}=wh+w_{\mathrm{gt}}h_{\mathrm{gt}}-W_{i}H_{i}$$

In the equation, IoU represents the Intersection over Union between the predicted box and the actual box. $W_{i}$ and $H_{i}$ denote the width and height of the intersecting rectangle between the actual box and the predicted box, while *w* and *h* represent the width and height of the predicted box, and $w_{\mathrm{gt}}$ and $h_{\mathrm{gt}}$ denote the width and height of the actual box. The original model’s loss function utilizes the CIOU [29]. The computation is as shown in Equation (8):

(8) $$L_{\mathrm{CloU}}=1-\mathrm{IoU}+\frac{{\left(x-x_{\mathrm{gt}}\right)}^{2}+\left(y-y_{\mathrm{gt}}\right)}{W_{g}^{2}+H_{g}^{2}}+\alpha v$$

(9) $$\alpha =\frac{v}{L_{\mathrm{IoU}}+v}$$

(10) $$v=\frac{4}{\pi^{2}}{\left(\arctan\frac{w}{h}-\arctan\frac{w_{\mathrm{gt}}}{h_{\mathrm{gt}}}\right)}^{2}$$

Within the formula, *v* denotes the aspect ratio consistency between the predicted box and the true box. $W_{g}$ and $H_{g}$ represent the width and height of the smallest enclosing rectangle around the actual and predicted boxes, while (x, y) indicates the center point of the predicted box, and ($x_{\mathrm{gt}}$, $y_{\mathrm{gt}}$) represents the center point of the actual box.

When the values of $\frac{w_{\mathrm{gt}}}{h_{\mathrm{gt}}}$ and $\frac{w}{h}$ are equal, ν=0, α and $L_{\mathrm{CIOU}}$ cannot be stably expressed. Moreover, the aspect ratio variation trend of CIOU is generally negatively correlated, easily causing target prediction box mismatch issues during boundary box prediction. The existing dataset lacks research on foreign objects in railway contact networks, and this experiment’s dataset is self-labeled, inevitably containing some low-quality labeled boxes. Therefore, we use the WIoU loss function [30] to optimize the model by introducing gradient gain and proposing a dynamic non-monotonic mechanism, focusing on ordinary anchor boxes. The computation is as shown in Equation (11):

(11) $$L_{\mathrm{WIoU}}=rR_{\mathrm{WloU}}L_{\mathrm{loU}}$$

(12) $$r=\frac{\beta }{\delta \alpha^{\beta -\delta }}$$

(13) $$R_{\mathrm{WloU}}=\exp\left(\frac{{\left(x-x_{\mathrm{gt}}\right)}^{2}+{\left(y-y_{\mathrm{gt}}\right)}^{2}}{\left(W_{g}^{2}+H_{g}^{2}\right)}\right)$$

(14) $$\beta =\frac{L_{\mathrm{loU}}^{*}}{\bar{L_{\mathrm{loU}}}}$$

Within the equation, α and β are hyperparameters, and δ is an adjustment parameter used to control the rate of change in weight *r*. To effectively prevent the generation of gradients that hinder convergence, the superscript “*” signifies the exclusion of Wg and Hg from gradient calculations. β represents the outlier degree, while $\bar{L_{\mathrm{IOU}}}$ signifies a dynamic variable. On one hand, this loss function assesses the quality of anchor boxes through the outlier degree, allocating smaller gradient enhancements when the value is significantly large or small, thereby diminishing the influence on bounding box regression. This approach enables the model to concentrate on anchor boxes of average quality to avoid excessive penalties due to geometric factors such as distance and aspect ratio. The refined loss function mitigates the detrimental gradients associated with extreme samples in vehicle detection—where the vehicle targets are exceedingly small or blurred—balances the model training outcomes across various image categories, and enhances the generalizability of the training results, leading to precise detection performance.

### 3.5. YOLOv8-PEL

**Figure 6:**
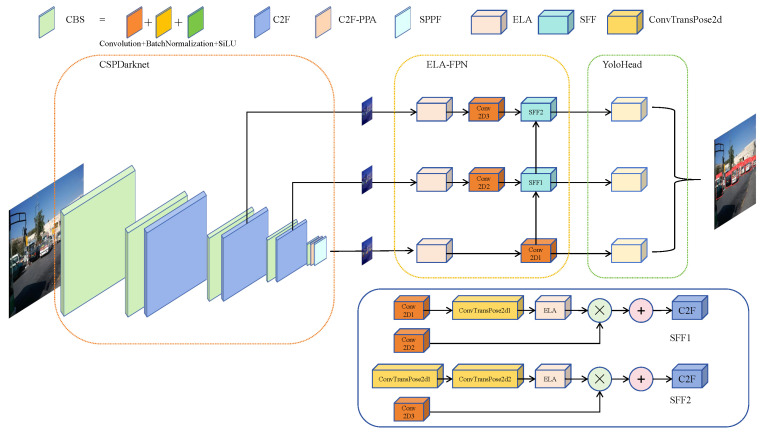
YOLOv8-PEL model structure.

Addressing the challenges of the YOLOv8n model in vehicle target detection tasks, the C2F-PPA module is utilized within the backbone network to preserve multi-scale features of the targets to be detected, thereby enhancing the model’s precision and generalization capabilities. At the feature fusion juncture, an augmented Feature Pyramid Network with Enhanced Local Attention is employed to bolster the capacity for feature selection and fusion, augmenting the efficacy of feature extraction, reducing the model’s size, and increasing its operational speed. This paper introduces the YOLOv8n-PEL, a lightweight vehicle detection model, the architecture of which is depicted in Figure 6.

## 4. The Dataset

We separated the images and labels corresponding to the three categories—car, bus, and truck—from the COCO object detection dataset [31] to create our training dataset, which we named the COCO-Vehicle dataset. This dataset contains 16,270 images in the training set and 707 images in the validation set. In the training set, the individual counts for the car, truck, and bus categories are 43,865, 9973, and 6069, respectively. In the validation set, the counts for these categories are 1932, 415, and 285, respectively. In the test set, the number of instances for cars, trucks, and buses is 5089, 1154, and 706, respectively. Additionally, we incorporated the VisDrone2019 dataset [32] to help analyze our model’s ability to detect small high-density objects. This dataset was collected by the AISKYEYE team at Tianjin University. It consists of 288 video clips, 261,908 frames, and 10,209 static images, with 6471 images in the training set, 548 in the validation set, and 3190 in the test set. The dataset was captured by various drone cameras, including images taken in 14 cities, covering a wide range of environments such as urban and rural areas, bright and dim lighting conditions, and various weather conditions. This diversity allows for a comprehensive evaluation of the model’s robustness across different backgrounds within controlled parameters. COCO-Vehicle is suitable for general vehicle detection and tasks related to ground vehicle detection, such as autonomous driving tasks. On the other hand, VisDrone2019 focuses on multi-object detection, high-density scenes, and small object scenarios from a drone perspective, making it suitable for tasks like traffic monitoring and drone navigation.

## 5. Experiments

The experiments in this study were conducted on the Ubuntu 20.04 operating system. The experimental environment included PyTorch 1.12.1, Python 3.8, and CUDA 12.2. The CPU was a 12th Gen Intel(R) Core(TM) i7-12700, and the GPU was an NVIDIA GeForce RTX 3090 with 24 GB of VRAM.

### 5.1. Model Evaluation Metrics

This study employs two categories of metrics: detection performance (Precision, Recall, mAP) and model efficiency (Params, GFLOPs, FPS).

1. Precision (Equation (15)) measures the proportion of true positives among predicted positives, reflecting false alarm suppression capability.
  (15) $$\mathrm{Precision}=\frac{\mathrm{TP}}{\mathrm{TP}+\mathrm{FP}}$$
2. Recall (Equation (16)) quantifies the coverage of actual positives, indicating missed detection avoidance.
  (16) $$\mathrm{Recall}=\frac{\mathrm{TP}}{\mathrm{TP}+\mathrm{FN}}$$
3. mAP (Equation (17)) evaluates multi-class detection robustness:
  (17) $$\mathrm{mAP}=\frac{1}{N}\sum_{i=1}^{N}{\mathrm{AP}}_{i}$$
  where mAP_0.5_ uses an IoU threshold of 0.5, while mAP_0.5:0.95_ averages precision across IoU thresholds from 0.5 to 0.95 (step 0.05).
4. Params: Model complexity indicator, balancing representation capacity (high params) against overfitting risks (low params).
5. GFLOPs: Computational complexity metric, critical for resource-constrained deployment.
6. FPS: Real-time processing capability, measured as processed frames per second.

### 5.2. Experiments Results

**Figure 7:**
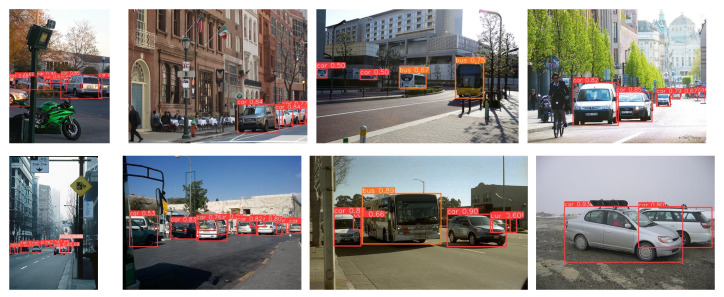
YOLOv8-PEL detection effect.

**Figure 8:**
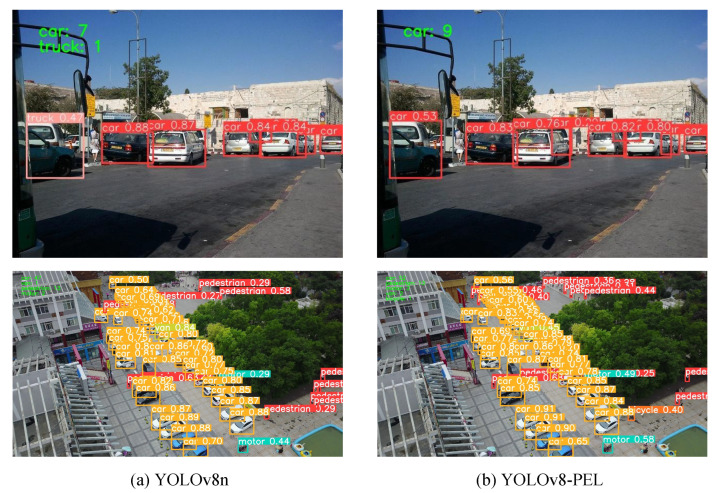
Comparison of detection performance between the original network model and the improved network model on the COCO dataset and VisDrone dataset.

**Figure 9:**
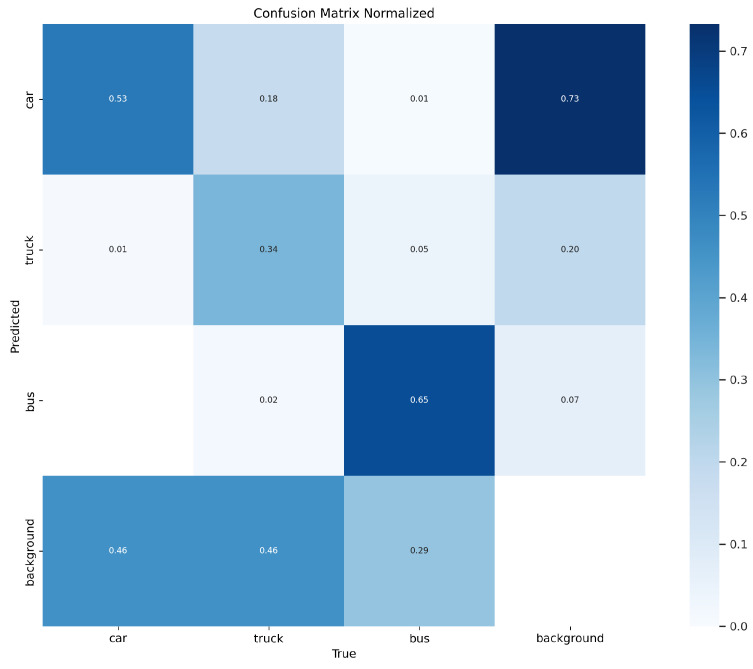
Confusion matrix normalized.

Figure 7 demonstrates that our model performs well in vehicle detection across various scenarios. Figure 8 shows a comparison of the inference results between our model and the original YOLOv8 algorithm on the COCO-Vehicle dataset and the VisDrone dataset. Figure 9 shows our confusion matrix, which illustrates the predictive distribution of the model across different categories.

Table 1 presents a comparison between our method and the original YOLOv8 algorithm on the COCO-Vehicle dataset. As shown in Table 1, although our model’s mAP decreased by 0.2%, the number of parameters was reduced by 30%, and the number of GFLOPs also decreased by 1.1. Therefore, our model effectively alleviates the issue of excessive hardware and software pressure on detection devices during vehicle detection. It not only improves computational efficiency but also reduces resource consumption during runtime, resulting in overall performance enhancement.

At the same time, we also focused on our model’s ability to detect objects in high-density scenarios with small targets. We conducted tests on the VisDrone dataset, as shown in Table 2. Our model demonstrated good performance, and while the mAP showed a slight increase, the number of parameters in our model decreased by 26% compared to YOLOv8n. This indicates that our model maintains a certain level of generalization while achieving lightweight design.

**Figure 10:**
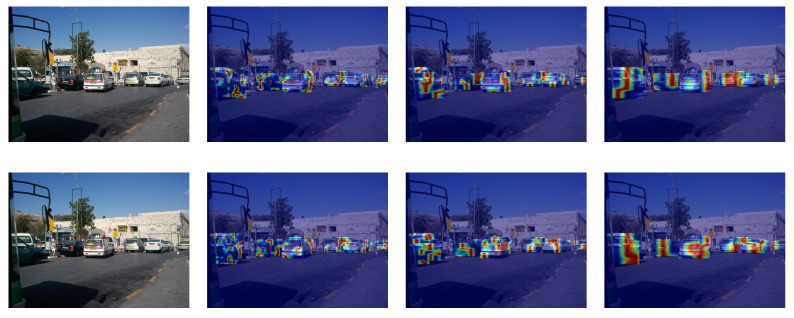
The feature maps extracted from CSPDarkNet53 (**top**) and the improved CSPDarkNet53 (**bottom**) are visualized. From left to right, the visualizations display the original image, heatmaps of feature 4, feature 6, and feature 9, respectively.

To validate the effectiveness of the proposed backbone, we visualized the fourth, sixth, and ninth layers of CSPDarkNet53 before and after the improvements, as these three layers serve as the input feature layers for the FPN. Figure 10 presents the visualization results of the feature extraction in the form of heatmaps. In the fourth and sixth layers, the original backbone retains more low-level texture information. However, in the final output feature map of the backbone (the sixth layer), the improved backbone with the C2F-PPA layers can generate more significant features, while the original backbone overlooks some valuable information.

In terms of the choice of loss functions, we also conducted experiments to compare the impact of various loss functions [33,34] on the model. As shown in Table 3, WIoU demonstrates the best performance. As shown in Table 4, we conducted ablation studies to demonstrate that replacing CA with ELA leads to significant performance improvements.

**Figure 11:**
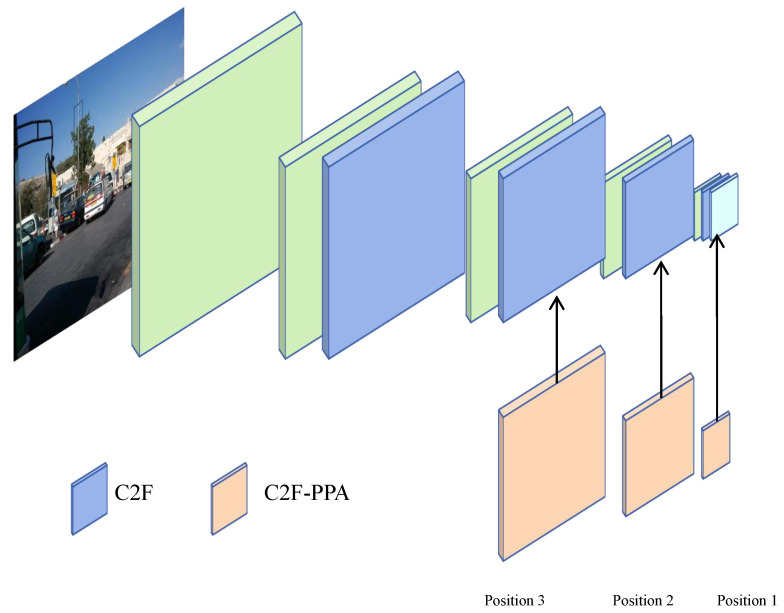
Different positions of C2F-PPA module in the Backbone.

Initial experiments replacing all C2F modules in CSPDarknet53 with C2F-PPA modules demonstrated suboptimal performance. To resolve this, we strategically integrated C2F-PPA modules into specific hierarchical layers [35] (denoted as Position 1, Position 2, Position 3 from bottom to top in Figure 11) to balance semantic richness and computational efficiency.

As quantified in Table 5, localized integration of C2F-PPA modules at Position1 (deepest layer) optimized semantic feature extraction, achieving a 66.9% mAP@0.5 with minimal parameters (2.23 M) and computational load (7.0 GFLOPs). Conversely, expanding replacements to shallow layers (Position 2 + 3) induced a 2.0% mAP@0.5 decline and 7.1% parameter growth. This degradation stems from excessive expansion of the receptive field and network depth, which suppressed geometric feature preservation in shallow layers—critical for small-to-medium target detection.

Therefore, restricting C2F-PPA to deep layers can not only utilize multi branch structures for advanced feature refinement, but also preserve shallow localization clues through limited depth scaling.

### 5.3. Ablation Experiments

To demonstrate the effectiveness of each proposed improvement module in enhancing vehicle object detection, we conducted ablation experiments for evaluation. The experimental results are shown in Table 6.

First, the baseline model YOLOv8n, without any improvement modules, exhibited relatively balanced performance, with a FPS of 165.7. On this basis, we replaced the C2f module in YOLOv8n with the designed C2F-PPA module. The experimental results showed that although the recall rate and FPS slightly decreased, the model’s precision increased from 71.6% to 72.3%, and mAP@0.5 also improved from 67.1% to 67.3%. The C2F-PPA module enhances feature extraction capability by introducing a multi-branch structure, similar to a dense residual structure. This design is not only easy to optimize but also improves detection accuracy by increasing network depth. Next, we introduced the ELA-FPN module to improve the model. The experimental results revealed that the model’s parameter count decreased from 3.01 M to 1.98 M, and the GFLOPs dropped from 8.1 to 6.9, significantly reducing the model’s complexity and computational requirements. Meanwhile, the inference speed increased to 182.6 FPS, a gain of 16.9 FPS. Although mAP@0.5 slightly decreased to 65.7%, ELA-FPN optimized the feature fusion structure, enhancing the model’s ability to handle multi-scale features, thereby significantly improving detection efficiency while reducing computational costs. Subsequently, we introduced the WIoU loss function. WIoU balances the training effects of different class samples by reducing harmful gradients from extreme samples during training, resulting in an increase in mAP@0.5 to 67.4%, a 0.3% improvement over the baseline model. At the same time, the inference speed increased to 169.3 FPS, a gain of 3.6 FPS. This improvement further optimized the stability of the training process and inference efficiency while maintaining model performance. Finally, we combined the C2F-PPA, ELA-FPN, and WIoU modules. The experimental results showed that the combined modules achieved an mAP@0.5 of 66.9%, close to the baseline model’s detection accuracy (67.1%), while the parameter count was reduced to 2.23 M, the computational cost decreased to 7.0 GFLOPs, and the inference speed increased to 176.8 FPS, a gain of 11.1 FPS compared to the baseline model. This combined improvement significantly enhanced inference efficiency while reducing model complexity.

Through the above improvements, compared to the baseline model YOLOv8n, the final YOLOv8-PEL model reduced the parameter count and computational cost by 0.78 M and 1.1 GFLOPs, respectively, significantly lowering the model’s complexity and improving efficiency. At the same time, the inference speed increased from 165.7 FPS to 176.8 FPS, further enhancing the model’s real-time detection capability. The results of the ablation experiments fully demonstrate the effectiveness of the proposed improvement modules in enhancing detection accuracy, achieving lightweight design, and improving inference speed, validating the feasibility and advantages of this method in the field of vehicle detection.

### 5.4. Comparison Experiment

To further validate the performance of YOLOv8-PEL, we compared its performance with several mainstream object detection models [36,37,38]. We focused on evaluating key metrics for each model, such as mAP@0.5, parameters, FLOPs, model size and FPS. The experimental results are shown in Table 7.

As shown in Table 7, YOLOv8-PEL demonstrates excellent performance in terms of model efficiency. With only 2.23 M parameters, 7.0 GFLOPs, a model size of 4.5 MB, and an FPS of 176.8, YOLOv8-PEL significantly outperforms the other models in the control group. Compared to the original YOLOv8n model, YOLOv8-PEL achieves an mAP@0.5 of 66.9%, with only a slight decrease of 0.2 in mAP@0.5, while the parameters, GFLOPs, and model size are reduced by 25%, 13%, and 25%, respectively. Additionally, the inference speed increases from 165.7 FPS to 176.8 FPS. In many cases, a slight reduction in accuracy in exchange for higher operational speed is a reasonable choice, especially in application scenarios with limited computational resources. This trade-off is acceptable, and the results confirm that YOLOv8-PEL can more effectively perform vehicle object detection tasks while maintaining a lightweight design.

## 6. Conclusions

To address the issue of low efficiency in vehicle detection on resource-constrained edge computing platforms, this paper proposes an enhanced YOLOv8-PEL model based on YOLOv8. The model introduces the C2F-PPA module, which employs a parallel multi-branch strategy to enhance multi-scale feature extraction capability, thereby improving detection accuracy and generalization performance. During the feature fusion process, an ELA-FPN is designed, optimizing feature selection and fusion capabilities through 1D convolution and GN. This significantly reduces the model’s parameter count and computational complexity while increasing operational speed. Additionally, the WIoUv3 loss function is introduced, which reduces the impact of extreme samples on the training process through a dynamic non-monotonic mechanism and gradient gain, enhancing focus on ordinary samples and further improving bounding box regression accuracy and detection efficiency. Experimental results demonstrate that YOLOv8-PEL achieves a good balance between detection accuracy and computational efficiency, making it suitable for deployment in real-time and resource-constrained environments. Although YOLOv8-PEL excels in vehicle detection tasks, its performance in complex environments (such as nighttime, rain, fog, and snow) still requires further improvement. Future work will focus on constructing larger-scale and more diverse scene datasets, combined with technologies such as infrared imaging, to further enhance the model’s robustness and adaptability in complex environments. At the same time, extensive field testing and practical application validation will contribute to the comprehensive optimization and promotion of the model.

## Figures and Tables

**Table 1:** Experimental results in COCO-Vehicle dataset. (The bold data in the table indicate the best results).

Methods	mAP_50_ (%)	Params	GFLOPs	Model Size
YOLOv8n	67.1	3.01 M	8.1	6.0 M
YOLOv8-PEL	66.9	**2.23 M**	**7.0**	**4.5 M**

**Table 2:** Experimental results in VisDrone dataset. (The bold data in the table indicate the best results).

Methods	mAP_50_ (%)	Params	GFLOPs	Model Size
YOLOv8n	32.3	3.01 M	8.1	6.0 M
YOLOv8-PEL	**32.5**	**2.23 M**	**7.0**	**4.5 M**

**Table 3:** Comparison IoU in COCO-Vehicle dataset. (The bold data in the table indicate the best results).

IoU Methods	P/%	R/%	mAP_50_ (%)
IoU	75.8	55.4	65.4
GIoU	69.4	**61.8**	65.5
EIoU	**76.1**	52.1	60.5
CIoU	71.6	61.3	67.1
WIoU	72.0	59.1	**67.4**

**Table 4:** Ablation Experiments of ELA and CA in COCO-Vehicle dataset. (The bold data in the table indicate the best results).

Methods	mAP_50_ (%)	Params	GFLOPs
YOLOv8n	**67.1**	3.01 M	8.1
+CA-FPN	65.2	2.03 M	7.2
+ELA-FPN	65.7	**1.98 M**	**6.9**

**Table 5:** The effect of replacing C2F with C2F-PPA modules at different locations in the Backbone network. (The bold data in the table indicate the best results).

Position	mAP_50_ (%)	Params	GFLOPs
Position 1 + 2 + 3	66.0	2.39 M	7.7
Position 1 + 2	64.9	2.36 M	7.3
Position 1	**66.9**	**2.23 M**	**7.0**

**Table 6:** Ablation Experiments in COCO-Vehicle dataset.

Model	P/%	R/%	mAP_50_ (%)	Params	GFLOPs	FPS
YOLOv8n (baseline)	71.6	61.3	67.1	3.01 M	8.1	165.7
+C2F-PPA	72.3	59.4	67.3 (+0.2)	3.25 M (+0.24)	8.2 (+0.1)	127.4 (−38.3)
+ELA-FPN	72.8	57.3	65.7 (−1.4)	1.98 M (−1.03)	6.9 (−1.2)	182.6 (+16.9)
+WIOU	72.0	59.1	67.4 (+0.3)	3.01 M (+0)	8.1 (+0)	169.3 (+3.6)
+C2F-PPA + ELA-FPN + WIOU	71.9	58.7	66.9 (−0.2)	2.23 M (−0.78)	7.0 (−1.1)	176.8 (+11.1)

**Table 7:** Comparison Experiments in COCO-Vehicle dataset.

Methods	mAP_50_ (%)	Params	GFLOPs	ModelSize	FPS
Faster R-CNN	**67.3**	41.39 M	208	167 MB	44.2
Cascade R-CNN	67.0	69.29 M	236	275 MB	32.7
RTMDet-tiny	65.6	4.88 M	8.1	9.3 MB	142.4
YOLOv3tiny	60.9	12.13 M	18.9	33.7 MB	163.8
YOLOv5n	66.6	2.50 M	7.1	5.0 MB	158.1
YOLOv6n	66.4	4.24 M	11.8	8.3 MB	172.2
YOLOv8n	67.1	3.01 M	8.1	6.0 MB	165.7
YOLOv10n	66.3	2.37 M	7.0	5.0 MB	152.6
YOLOv8-PEL	66.9	**2.23 M**	**7.0**	**4.5 MB**	**176.8**

## Data Availability

The original contributions presented in this study are included in the article. Further inquiries can be directed to the corresponding author.
